# Stigmatization as a barrier in opioid substitution therapy patients

**DOI:** 10.1192/j.eurpsy.2021.2166

**Published:** 2021-08-13

**Authors:** M.J. Gonçalves, C. Sereijo, R. André, G. Andrade, R. Saraiva, L. Linhares, I. Chendo, M. Abreu

**Affiliations:** 1 Psychiatry, Centro Hospital Lisboa Norte, Lisboa, Portugal; 2 Psiquiatria, Centro Hospitalar Universitario Lisboa Norte, oeiras, Portugal; 3 Psiquiatria, Centro Hospitalar Universitario Lisboa Norte, Lisboa, Portugal

**Keywords:** methadone, opioid use disorder, Stigma, opioid substitution therapy

## Abstract

**Introduction:**

Goffman defined stigma as an “attribute that is deeply discrediting” and in the last two decades research on this subject grew substantially.Opioids were ranked as the second most common form of illicit drug used worldwide and there is consensus in the literature that opioid substitution therapy (OST), methadone or buprenorphine, are the most effective treatments, although remain underutilized. People with an history of substance use disorders (SUD) are widely stigmatized, a significant barrier to detection and treatment efforts. Care workers were cited as the second most common source of stigma.

**Objectives:**

The aim is to do a review of the literature of stigma as a significant barrier to OST and present several potential strategies to reduce stigma.

**Methods:**

Non-systematic review of the literature with selection of scientific articles published in the last 5 years; by searching the Pubmed and Medscape databases using the combination of MeSH descriptors. The following MeSH terms were used: Opioid Use Disorder; Stigma; Opioid Substitution Therapy

**Results:**

OST providers should actively bring up the topic of stigma in clinic appointments to determine whether the patient is experiencing stigma, and if so, whether it is adversely affecting their ability to continue in the treatment. More active measures need to be taken to help reducing the stigma through public awareness campaigns at local levels, continuing education of health care providers regarding substance OST, and greater incorporation of family members into the program.

**Conclusions:**

In conclusion, further research is required to understand and address this issue.

**Disclosure:**

No significant relationships.

